# Virtual shared medical appointments for obesity and GLP-1 medications: A scalable method for optimizing care delivery

**DOI:** 10.1016/j.obpill.2026.100277

**Published:** 2026-05-20

**Authors:** Jacob Mirsky, Olayemi Olubowale, Ryan M. Kane

**Affiliations:** a Department of Medicine, Massachusetts General Hospital, Boston, MA, USA; b Harvard Medical School, Boston, MA, USA; c Massachusetts General Hospital Weight Center, Boston, MA, USA; d Division of General Internal Medicine, Department of Medicine, Tufts University School of Medicine, Boston, MA, USA; e Food is Medicine Institute, Friedman School of Nutrition Science and Policy, Tufts University, Boston, MA, USA

**Keywords:** GLP-1 medications, Obesity, Shared medical appointment, Telemedicine

## Abstract

**Background:**

More than half of U.S. adults are eligible for glucagon-like peptide-1 receptor agonist and multi-target incretin (GLP-1) medications. Obesity guidelines recommend multicomponent intensive lifestyle change programs in conjunction with GLP-1 medications; however, there is limited health system infrastructure for this recommended programming.

**Methods:**

This was a single site, retrospective cohort study of patients who attended ≥1 virtual shared medical appointment (SMA) in a 4-part series on obesity and GLP-1 medications at the Massachusetts General Hospital (MGH) Healthy Lifestyle Program from October 2024 to September 2025. Included are descriptive assessments of patient engagement, prescriptions, and weight loss among a sub-group of highly engaged patients who attended ≥3 virtual SMAs and began the series below the maximum GLP-1 dose.

**Results:**

Of 54 enrolled patients from 5 cohorts, 33 patients completed ≥1 virtual SMA. The mean (standard deviation, SD) age was 58.0 years (12.8) and 24 (73%) patients were female. In the sub-group of 20 patients who started the series below the maximum GLP-1 dose and attended ≥3 virtual SMAs, 14 (70%) had a dose increase, 1 (5%) had a dose decrease, 4 (20%) had no change in dose, and 1 (5%) switched medications; no patients discontinued GLP-1 medication therapy during the series. There were 17 patients from the sub-group who had weight data in the electronic medical record within 6 months of both the start and end of the series. Pre-and post-series mean (SD) weight was 227 lb (53 lb) and 211 lb (46 lb), respectively; weight decreased by 0–5% for 7 (41%) patients, 6–10% for 6 (35%) patients, and ≥10% for 4 (24%) patients.

**Conclusion:**

The main finding was that attendance in the virtual SMA series, in a sub-group of highly engaged patients who attended ≥3 virtual SMAs and began the series below the maximum GLP-1 dose, was associated with GLP-1 medication dose increases in 70% of participants and all participants remained on GLP-1 medication. Virtual SMAs hold promise for increasing the scalability and efficacy of GLP-1 medication use in conjunction with intensive lifestyle counseling for obesity in primary care.

## Graphical abstract

**Figure:**
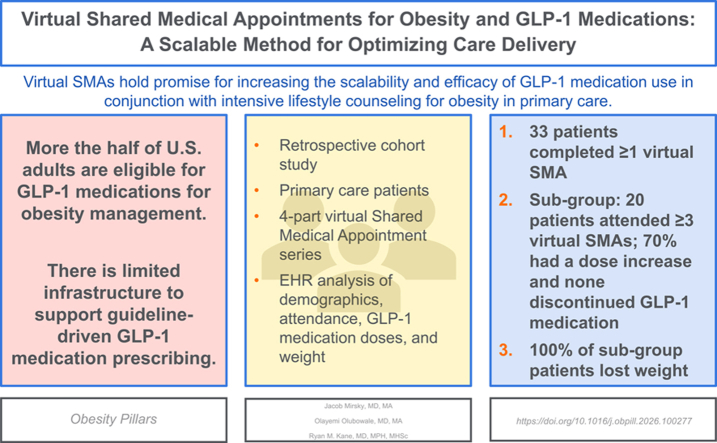

## 1 Introduction

More than half of U.S. adults are eligible for glucagon-like peptide-1 receptor agonist and multi-target incretin (referred to hereafter as “GLP-1”) medications [1,2]. The rapid rise of GLP-1 medications has exacerbated several challenges for obesity management. For example, obesity guidelines recommend multicomponent intensive lifestyle change programs in conjunction with GLP-1 medications; however, there is limited health system infrastructure for this recommended programming [3]. As such, GLP-1 medications are often prescribed without critical supports, resulting in low long-term adherence, which increases the risk for harms and limits their potential clinical benefits and cost-effectiveness [[2], [3], [4]]. This lack of guideline-directed care and limited real-world efficacy is particularly concerning as GLP-1 medication use for obesity may rise with increasing affordability through federal price negotiations [5].

Virtual shared medical appointment (SMA) programming focused on GLP-1 medication use for optimal obesity management offers hope in addressing some of these challenges. Virtual SMAs can bring multiple patients together with one or more clinicians and incorporate lifestyle recommendations, side effect management, and medication titration in billable visits [6]. We sought to evaluate the feasibility and explore the impact of GLP-1 medication-focused virtual SMAs on patient engagement, prescriptions, and weight loss.

## 2 Methods

This retrospective cohort study was guided by the Learning Health System Framework to optimize adaptable and accessible health system care delivery to promote equitable, effective, and efficient use of evidence-based therapies, such as GLP-1 medications [7].

The Massachusetts General Hospital (MGH) Healthy Lifestyle Program started virtual SMAs in 2020 and launched a GLP-1 medication-focused series in October 2024 [8]. MGH primary care patients who initiated GLP-1 medications within 6 months of the first virtual SMA session were eligible to self-refer or were referred by their primary care providers (PCPs). These virtual SMAs were initially open to any MGH Primary Care patient with obesity and any GLP-1 medication use and were narrowed to patients using GLP-1 medications specifically for obesity due to high demand.

In line with standard workflows in the Healthy Lifestyle Program, a maximum of 12 patients were scheduled in each series, which consisted of 4 60-min SMA sessions held once every 2 weeks. Each session consisted of approximately 30 min focused on didactics and 30 min dedicated to individualized check-ins with each patient by a PCP and a health and wellness coach.

In this series, the topics covered in each session included 1) GLP-1 medication use, benefits, and side effects; 2) plant-based nutrition emphasizing fiber and protein intake; 3) regular cardiovascular and muscle-strengthening activities; and 4) the positive health benefits of sleep, stress reduction, and positive psychology. Patients were encouraged to make behavior change goals in each session. Additionally, GLP-1 medication doses were titrated based on clinical judgment (i.e., there was no standardized protocol). The cohort included all patients who attended ≥1 virtual SMA between October 2024 and September 2025 with a sub-group of highly engaged patients who attended ≥3 virtual SMAs and began the series below the maximum GLP-1 medication dose. All data were reported descriptively for this exploratory analysis. Data collection included patient demographics, virtual SMA attendance, and GLP-1 medication prescriptions. Weight changes were assessed using the most proximal entries from the electronic medical record in the 6 months before and after the series. The study was reviewed and deemed exempt by the Mass General Brigham Institutional Review Board. This manuscript followed the Strengthening the Reporting of Observational Studies in Epidemiology (STROBE) statement: guidelines for reporting observational studies [9].

## 3 Results

Over 5 cohorts of 54 enrolled patients, 33 patients (61%) completed ≥1 virtual SMA. Of these 33 patients, the mean (standard deviation, SD) age was 58.0 years (12.8) and 24 (73%) were female. There were 22 (67%) patients on commercial insurance and 11 (33%) patients on Medicare/Medicare Advantage. Patients started the series on tirzepatide (n = 24, 72%), semaglutide (n = 8, 25%), and dulaglutide (n = 1, 3%); it is not known whether medications were covered by insurance. Patients who did not complete a virtual SMA, as part of usual care, were not automatically rescheduled into a future series.

**Fig. 1:**
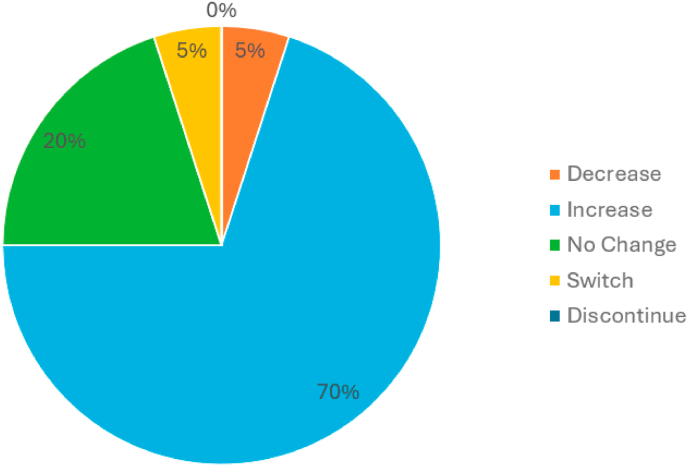
GLP-1 medication changes during the virtual Shared Medical Appointment (SMA) series for patients who attended at least 3 sessions and who started the series below the maximum dose.

**Fig. 2:**
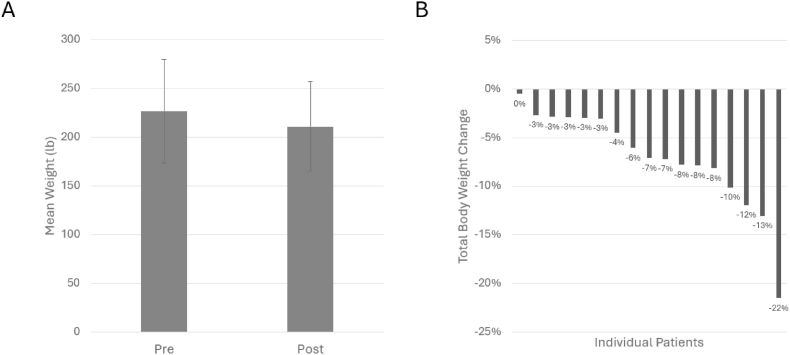
Mean (A) and individual (B) patient changes in total body weight in the 6 months before and after a virtual Shared Medical Appointment (SMA) series for patients who attended at least 3 sessions and who started the series below the maximum GLP-1 medication dose.

Twenty patients started the series below the maximum GLP-1 dose and attended ≥3 virtual SMAs. Of these 20 patients, 14 (70%) had a dose increase, 1 (5%) had a dose decrease, 4 (20%) had no change in dose, and 1 (5%) switched medications (Fig. 1). Within this sub-group, no patients discontinued GLP-1 use during the series. There were 17 patients from the sub-group (85%) who had weight data pre- and post-series (mean interval between measurements: 124 days). Pre-and post-series mean (SD) weight was 227 lb (53 lb) and 211 lb (46 lb), respectively (Fig. 2). All 17 patients (100%) lost weight. The mean percent total body weight change was 7% (16 lb); weight decreased by 0–5% for 7 (41%) patients, 6–10% for 6 (35%) patients, and ≥10% for 4 (24%) patients.

## 4 Discussion

This single-arm retrospective cohort study highlights the potential for GLP-1 medication-focused virtual SMAs to enhance access to comprehensive, guideline-concordant obesity care that can support PCPs’ management of GLP-1 medication titration—a key factor for maximizing the therapeutic effects of GLP-1 medications in practice [10]. Specifically, this study suggests that virtual SMAs focused on GLP-1 medication titration for obesity management are feasible in an academic primary care setting.

Given the concerns over high GLP-1 medication discontinuation rates [3], we focused our descriptive analyses on the highly engaged patients with the most potential for benefit (i.e., those who started the series below the maximum dose) to explore the potential for patient engagement and clinical improvements associated with virtual SMA program participation. Within this sub-group, it is encouraging that no patients who attended ≥3 virtual SMAs discontinued GLP-1 use. In fact, 70% of patients in the highly engaged sub-group who entered the series below the maximum dose increased their GLP-1 doses, which likely drove the weight loss seen among all patients in the sub-group.

## 5 Limitation

This study's small size and the absence of a comparator group limits the ability to provide robust statistical assessments of the effectiveness of virtual SMAs to optimize GLP-1 use when compared to standard care. However, the positive exploratory findings of descriptive statistics are encouraging, especially for highly engaged patients. Additionally, GLP-1 medication changes were only evaluated within the series, which may lead to an underestimate of discontinuation rates over a longer timeframe. Future studies on long-term GLP-1 medication use, as well the financial sustainability of this model, will also be critical.

Prior work with similar virtual SMA programs has shown that participant interest in the content, group positivity, and session logistics (e.g., session timing and cadence) were important factors that facilitated participant engagement [6,11]. For future improvements to this virtual SMA model, it will be critical to identify reasons for low engagement (i.e., 61% attendance to at least 1 SMA session). A recent analysis of the MGH Healthy Lifestyle Program similarly found that 53% of all scheduled SMA sessions (for a wide range of chronic disease topics) were attended by patients, however only 21% of patient referrals resulted in an attended session [12].

## 6 Conclusion

GLP-1 medications are often prescribed without critical supports, resulting in low long-term adherence, which increases the risk for harms and limits their potential clinical benefits and cost-effectiveness. In this retrospective cohort, among patients who participated in 3 or more virtual SMAs and were below the maximum GLP-1 medication dose at baseline, most underwent dose escalation and remained on therapy during the observation period. These findings are descriptive and limited by selection bias and the lack of a comparator group.

• Primary care patients participated in virtual SMA programming for GLP-1 medications for obesity management.
• Virtual SMAs for GLP-1 medication titration, side-effect management, and comprehensive lifestyle change may offer a scalable solution to our current gaps in providing guideline-concordant obesity care.
• Future longitudinal trials of virtual SMAs in a variety of primary care contexts (e.g., initiation, maintenance, and discontinuation of GLP-1 medications) will be needed to explore the potential patient, clinical, operational, and financial benefits of virtual SMAs for obesity management.

## Author contribution

The concept of the submission was by JM and RMK. Statistical analysis and Data Curation was performed by JM. JM and RMK wrote the first draft. JM, OO, and RMK all reviewed, edited, and approved the final submission and publication.

## Disclosures

Dr. Mirsky reports prior funding from the Ardmore Institute of Health and the Vitamix Foundation; he is also the founder and owner of Lifestyle Medicine Consulting LLC and SMA Catalyst. Dr. Olubowale has no relevant disclosures to report. Dr. Kane reports prior funding from the Ardmore Institute of Health.

## Ethics

The study was reviewed and deemed exempt by the Mass General Brigham Institutional Review Board.

## Artificial intelligence

During the preparation of this work the author(s) did not use AI.

## Funding

No funding was received for this research.
